# From surface terminations to functional nanoarchitectures: a chemistry-first framework for stabilizing and programming MXenes

**DOI:** 10.3389/fchem.2026.1911996

**Published:** 2026-07-28

**Authors:** Haotian Wu, Yinxu Xie, Shengjun Ji, Bangsheng Yin

**Affiliations:** College of Mechanical Engineering, Shandong Huayu University of Technology, Dezhou, Shandong, China

**Keywords:** chemical sensing, interface engineering, ion transport, MXene, nanoarchitecture, oxidation stability, surface terminations

## Abstract

MXenes combine metallic conductivity, solution processability, and chemically addressable surfaces, but the reactive interfaces that enable functionalization also accelerate oxidation, restacking, and property drift. This Mini Review develops a chemistry-first framework in which surface terminations, defects, adsorbates, and interlayer species are treated collectively as a coupled state variable rather than as independent descriptors. We link etching and delamination chemistry to termination populations and discuss the effects of water and oxygen on spatially heterogeneous degradation. We then examine how molecular ligands, polymers, inorganic phases, and mesoscale assembly modulate interfacial reactions and transport pathways. We organize recent studies according to a causal sequence: chemical intervention, nanoscale structural consequence, transport response, functional output, and failure mode. This sequence helps explain why nominally similar Ti_3_C_2_T_x_ materials can exhibit divergent electrochemical, catalytic, sensing, mechanical, and electromagnetic behavior. We argue that further progress depends less on identifying isolated applications than on controlling and reporting the evolving interfacial state across synthesis, storage, processing, and operation. We propose testable design rules for termination-aware synthesis, kinetic stabilization, interface-selective assembly, and operando validation. This framework positions MXenes as programmable reactive nanoarchitectures and identifies reproducibility, scalable fluorine-lean chemistry, and state-resolved characterization as priorities for nanoscience.

## 1 Introduction

MXenes are two-dimensional transition-metal carbides, nitrides, and carbonitrides obtained by selectively removing an A-layer from layered MAX or related precursors (Naguib et al., 2011). Their combination of high electronic conductivity, hydrophilic surfaces, nanometer-scale thickness, and dispersibility has made them a versatile platform for electrochemical storage, catalysis, sensing, separations, electromagnetic control, and biomedical interfaces (Anasori et al., 2017). The field has consequently expanded beyond what can be adequately captured by a single application-centered review. However, this versatility presents a persistent paradox: the exposed surfaces that make MXenes chemically programmable are also responsible for oxidation, ion-specific swelling, restacking, and irreversible changes during storage and operation (Anasori et al., 2017; Halim et al., 2016). Processing guidelines, X-ray photoelectron spectroscopy, and termination-sensitive calculations collectively show that the formula M_n+1_X_n_T_x_ is incomplete. T_x_, defects, intercalants, hydration, and oxidation products must also be considered explicitly (Alhabeb et al., 2017; Halim et al., 2016; Hart et al., 2019). Consequently, two samples labeled Ti_3_C_2_T_x_ may not be chemically or functionally equivalent.

This Mini Review advances a focused proposition: MXene performance emerges from an evolving interfacial chemical state. Etching determines vacancies and initial terminations, while washing and delamination select counterions and hydration structures. Storage, functionalization, assembly, and operation then reshape oxidation, charge density, wettability, transport, stress, and local heating. Although often discussed separately, these steps form one reaction-processing chain. Reviews of synthesis, environmental stability, and interface engineering have documented many components of this chain (Iqbal et al., 2021; Li et al., 2019; Palei et al., 2023). What remains missing is a causal framework that connects a controllable chemical intervention to a structural consequence, then to transport, function, and failure.

**FIGURE 1:**
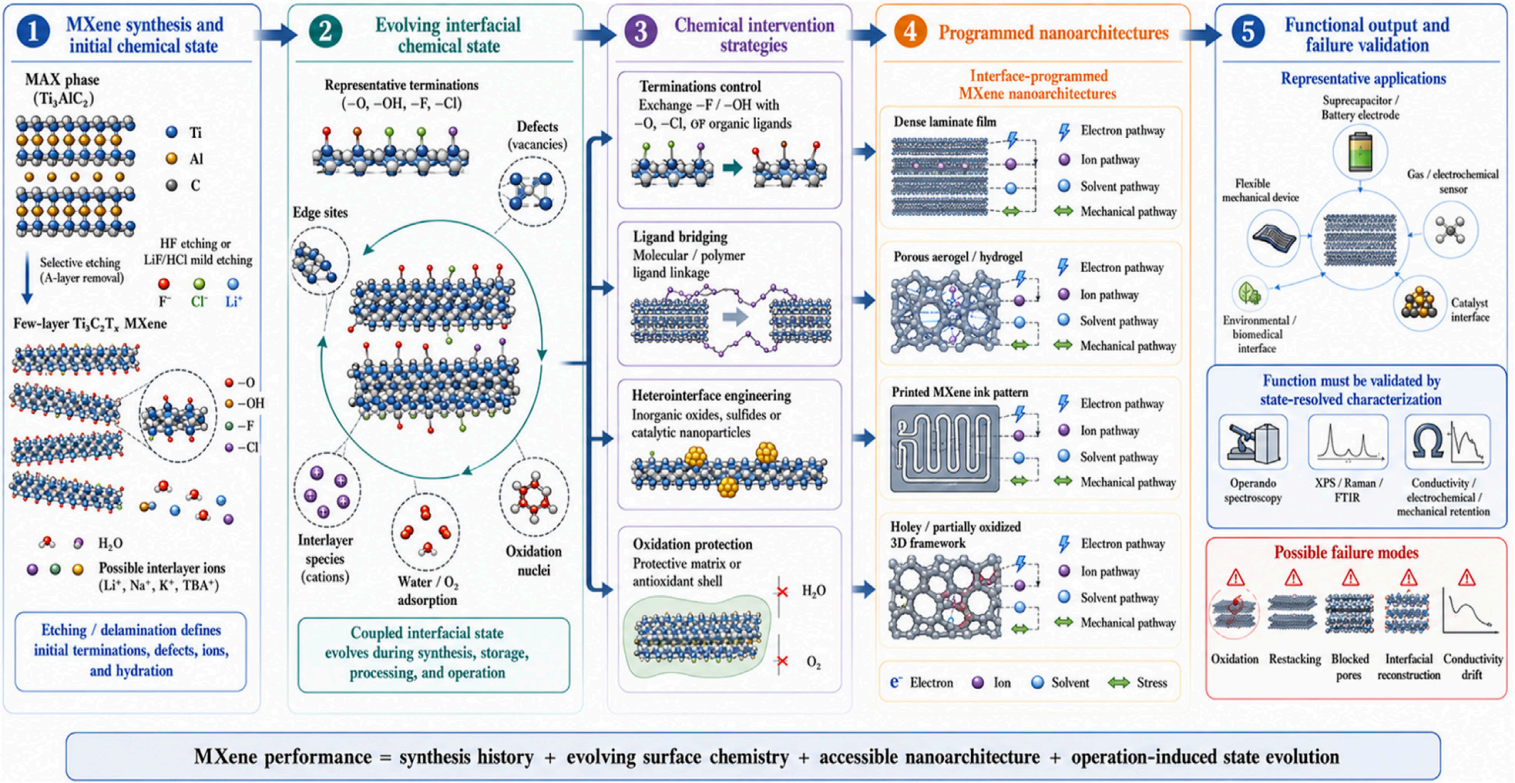
Chemistry-first framework linking MXene synthesis history to an evolving interfacial chemical state, targeted chemical intervention, programmed nanoarchitectures, and function/failure validation. Selective etching and delamination define terminations, defects, ions, hydration, and edge chemistry, which continue to evolve during storage, processing, and operation. Termination control, ligand bridging, heterointerface engineering, and oxidation protection redirect these variables into dense, porous, printed, or partially oxidized architectures that regulate electron, ion, solvent, and mechanical pathways. State-resolved characterization is required to distinguish functional gains from oxidation, restacking, pore blockage, interfacial reconstruction, and conductivity drift. The atomic-scale and nanoscale drawings in this figure are idealized schematic representations intended to illustrate surface-state concepts and reaction–transport relationships rather than quantitatively resolved crystallographic models.

This framework is relevant to chemistry-focused nanomaterials research because it emphasizes synthesis, assembly, characterization, and chemical control of nanoscale structures rather than catalogs of applications. We concentrate on Ti_3_C_2_T_x_ because it has the most extensive experimental evidence, while we use other MXenes to expose composition-dependent behavior. The discussion integrates foundational studies with recent research on termination exchange, nontraditional etching, degradation pathways, covalent modification, hybrid interfaces, printing, porous architectures, and devices (Adomaviciute-Grabusove et al., 2024; Huang and Han, 2023; Liu L. et al., 2022; Zhou et al., 2023). Our central premise is that surface chemistry is not merely one optimization parameter, but the principal variable linking nanoscale identity to macroscopic function. Because of the Mini Review format, the mechanistic discussion focuses on representative reaction motifs, including edge/defect-initiated oxidation, termination/intercalant-controlled solvation, ligand passivation or bridging, and controlled partial conversion, rather than exhaustive elementary reaction networks. Figure 1 summarizes this state-evolution sequence, from synthesis and chemical intervention through nanoarchitecture formation to functional output and failure validation.

## 2 A coupled chemical state created during synthesis

The interfacial state is established before a free-standing sheet is formed. Conventional fluoride-containing routes remove Al from Ti_3_AlC_2_ while generating mixtures of–F, –OH, and–O terminations. Acid concentration, fluoride activity, temperature, time, precursor grain size, and post-etch washing affect both removal kinetics and defect formation. The widely used LiF-HCl route couples etching with Li-containing intercalation, which facilitates delamination but leaves a distinct ionic and hydration history. Processing studies demonstrate that centrifugation, sonication, dissolved oxygen, and flake size affect product yield and quality; therefore, a synthesis label alone cannot define the product (Alhabeb et al., 2017). Lewis-acidic molten salts broaden the range of accessible compositions and can install halogen terminations, while subsequent Lewis-basic halides can tune both interlayer spacing and surface groups (Huang and Han, 2023; Liu L. et al., 2022; Zhang et al., 2022). Together, these routes show that selective etching and termination chemistry are inseparable reactions. This coupling becomes more actionable when one synthesis variable is changed while the parent chemistry is otherwise constrained. For example, etchant-dependent MXene synthesis studies show that changing the fluoride salt can alter cation intercalation or hydration, gallery spacing, flake morphology, charge-transfer resistance, and capacitance, indicating that the etchant should be treated as a chemical-state variable rather than as a neutral Al-removal reagent (Ghorbanzadeh et al., 2026).

Termination populations reshape the local electronic structure by withdrawing or donating charge, modifying the work function, and changing adsorption energies. First-principles and experimental comparisons indicate that surfaces rich in–O, –OH, and–F groups are not interchangeable. Termination-dependent studies show that surface groups can shift work function, carrier density, adsorption strength, and charge-transfer behavior, while recent high-throughput calculations across idealized single-termination MXenes further link termination identity to work function and surface charge. These calculations are useful reference states, but they should not be treated as direct descriptions of experimentally mixed Ti_3_C_2_T_x_ surfaces (Ghorbanzadeh and Zhang, 2025; Hart et al., 2019). They alter metallicity, carrier density, magnetic tendencies, and affinity for ions or molecules (Hart et al., 2019; Portugal and Rosen, 2025). Hybrid organic-inorganic MXenes with amido and imido terminations clearly demonstrate that surface replacement can create chemical identities inaccessible through conventional aqueous etching (Zhou et al., 2023). Salt-melt nitrification similarly improves specific storage responses by changing terminal atoms rather than merely depositing a coating (Liu et al., 2023). The relevant descriptor is therefore not simply the nominal fraction of a single termination, but a spatial distribution coupled to vacancies, adsorbed water, counterions, and local oxidation state. This point is illustrated by processing and degradation studies showing that sonication, centrifugation, dissolved oxygen, and flake size alter product quality and oxidation behavior, while Raman monitoring reveals that local oxide formation can precede global loss of the MXene lattice (Adomaviciute-Grabusove et al., 2024; Alhabeb et al., 2017).

A second interaction links termination chemistry and colloidal behavior. Surface charge controls electrostatic repulsion, while termination-dependent hydrogen bonding and cation coordination control swelling and aggregation. Processing studies confirm that washing and delamination change wetting and dispersion, but macroscopic measurements average over roughness, contamination, and chemical heterogeneity (Alhabeb et al., 2017). Delamination produces smaller flakes and more edge sites, which improves dispersion and ink formation but increases the density of oxidation-prone regions. Conversely, large flakes support strong continuous films but can restack into transport-limited laminates. The practical objective is therefore not maximal delamination, but a target flake-size and surface-state distribution matched to the intended assembly route. Room-temperature printing of wireless electronics illustrates the need to balance rheology, conductivity, and flake integrity rather than optimizing any one variable independently (Shao et al., 2022).

Reproducibility requires a minimum set of chemical-identity descriptors. Performance data should be accompanied by a defined set of synthesis and state descriptors. These include precursor composition, etchant system, etching conditions, washing endpoint, delamination method, storage atmosphere, flake-size distribution, interlayer spacing, and termination/oxidation metrics. XPS is indispensable but should be supported by complementary diffraction, vibrational spectroscopy, elemental analysis, microscopy, and electrochemical or thermal probes. A Fourier-transform infrared library can improve assignment consistency, whereas Raman monitoring reveals time-dependent degradation pathways that cannot be captured by a single endpoint spectrum (Adomaviciute-Grabusove et al., 2024; Parker et al., 2024). Reporting a chemically resolved history turns synthesis from a recipe into a reproducible state-preparation protocol.

## 3 Oxidation is a reaction network, not a shelf-life number

Oxidation is commonly described by the time required for a dispersion to change color or lose conductivity. This metric obscures the reaction network. Water, dissolved oxygen, light, heat, pH, defects, edges, and transition-metal composition jointly govern oxidation nucleation and propagation. Mechanistic studies of water attack indicate that water is not an inert dispersant: it participates in surface reactions and can promote conversion into titanium oxides and carbonaceous residues (Wu et al., 2022). Solvent studies show that oxidation rates depend strongly on the surrounding medium and on whether flakes are dispersed or immobilized in a composite film (Habib et al., 2019). The apparent stability of a bulk film may therefore arise from diffusion limitation rather than intrinsically passivated surfaces.

At the molecular level, this degradation can be viewed as a coupled access–reaction–propagation sequence. Water and dissolved oxygen first reach edges, atomic defects, vacancies, and other locally under-coordinated regions where metal coordination is less saturated than on extended basal planes (Xia et al., 2019). Local hydrolysis and proton-coupled oxidation can then generate Ti–O/Ti–OH-rich species, which develop into oxide nuclei and carbonaceous residues (Wu et al., 2022). Once formed, these oxide-rich regions alter local wetting, electronic percolation, and charge-transfer pathways, thereby promoting spatially heterogeneous propagation rather than uniform basal-plane conversion (Adomaviciute-Grabusove et al., 2024).

This spatial heterogeneity is a central consideration for stability assessment. Edges, pinholes, vacancies, and strained regions provide low-barrier reaction sites; oxidation products then modify local wetting and electron transfer, and may thereby accelerate neighboring reactions. Smaller flakes increase edge-to-area ratio, whereas aggressive sonication can add defects that shorten diffusion distances for oxidants. Raman pathway monitoring and microscopy support a pathway in which local oxide formation precedes global loss of the MXene lattice (Adomaviciute-Grabusove et al., 2024). This explains why average XPS composition may appear deceptively acceptable while device resistance or electrochemical behavior has already drifted. Stability should therefore be evaluated using time-resolved distributions, not only initial and final averages. For this reason, oxidation should be reported through measurable outputs rather than only as a general loss of stability. Useful descriptors include the appearance and growth of oxide-related Raman features, loss of carbide-related spectral signatures, Ti–O contributions in surface spectra, conductivity decay, flake fragmentation, and changes in electrochemical response. Such reporting clarifies whether a stabilizing strategy delays oxide nucleation, slows oxide growth, preserves electronic percolation, or merely masks degradation by diffusion limitation (Adomaviciute-Grabusove et al., 2024; Wu et al., 2022).

Stabilization strategies fall into three mechanistic classes. Thermodynamic stabilization changes the surface state to make oxidation less favorable, for example through termination exchange or covalent ligands. Kinetic stabilization restricts oxygen, water, photons, or heat by low-temperature storage, deoxygenated solvents, dense films, antioxidants, or barrier matrices. Sacrificial stabilization uses a secondary phase to react preferentially with or neutralize reactive species. Green reduction routes have improved conductivity and oxidation resistance, while extracted bentonite in composites has enabled remarkable high-temperature stability in air (Limbu et al., 2020; Liu N. et al., 2022). These mechanisms should be distinguished because a barrier that delays oxidation under storage may fail during electrochemical swelling, and a ligand that protects basal planes may leave edges exposed.

The resulting design principle is to match the protection mechanism to the operational stressor. Each application imposes a distinct combination of stresses. Printed conductors must tolerate humidity and bending, supercapacitors must withstand ion insertion, catalysts must retain active sites, and biosensors must preserve recognition chemistry in complex fluids. Accelerated testing should therefore reproduce the coupled chemical and mechanical environment rather than relying on heat alone. A useful stability report includes retained conductivity or capacitance, chemical-state evolution, morphology, and the dominant failure pathway. Only then can storage lifetime be meaningfully related to operational lifetime.

## 4 Interface programming from molecules to nanoarchitectures

Molecular functionalization provides the most direct means of separating desirable reactivity from uncontrolled degradation. Small ions can regulate spacing and screen surface charge; zwitterions can increase electrolyte affinity while suppressing collapse; polymeric ligands can form covalent or multidentate contacts that redistribute stress. Zwitterionic surface nanoengineering produced a broad capacitance enhancement by changing interfacial solvation and accessibility rather than simply adding active mass (Děkanovský et al., 2023). Covalent modification with chemically active polymeric ligands generated conductive, ordered films, illustrating that strong bonding need not destroy electron transport when ligand density and conjugation are controlled (Lee et al., 2021). The key criterion is whether a modifier creates accessible and reversible interactions rather than an insulating, diffusion-blocking layer.

These interventions act at different mechanistic points in the degradation and transport sequence. Zwitterions and intercalated ions mainly tune solvation, electrostatic screening, and gallery swelling (Děkanovský et al., 2023); covalent or multidentate ligands occupy reactive surface sites and create stronger flake–flake or flake–polymer contacts (Lee et al., 2021); inorganic barrier phases reduce oxygen and water access (Liu N. et al., 2022), while controlled partial oxidation can arrest conversion at an intermediate state to generate holey or oxide/carbide architectures (Sikdar et al., 2024). In this sense, interface programming does not simply add a secondary component, but changes which elementary step becomes rate-limiting: oxidant access, surface reaction, charge transfer, ion diffusion, or mechanical failure.

Interfacial bridges are particularly valuable because pristine lamellae face a mechanical-transport trade-off. Strong attractive interactions increase cohesion but encourage restacking; open galleries improve diffusion but weaken load transfer. Ultrastrong films assembled from small intercalating flakes and interfacial bridges partly resolve this conflict by using different structural elements for gap filling and stress transfer (Wan et al., 2022). Bacterial cellulose offers a complementary multiscale strategy: its fibrous framework separates MXene sheets, creates microporosity, and supports flexible electrodes whose performance can be tuned through functional-group regulation (Luo et al., 2024). These examples show that the best interface is rarely the strongest possible interface; it is an interface with the correct bond lifetime, density, and spatial location.

Inorganic heterointerfaces introduce band alignment, catalytic sites, polarization centers, or mechanical contrast. Work-function engineering in Bi_2_S_3_/Ti_3_C_2_T_x_ accelerated photo-excited antibacterial action by directing charge transfer across the heterojunction (Li et al., 2021). MXene-metal oxide electrocatalysts similarly exploit conductive support, altered adsorption energetics, and interfacial reconstruction during oxygen evolution (Lakhan et al., 2024). Attributing activity is challenging because MXene oxidation can generate catalytically active oxides *in situ*. Claims of synergy should therefore differentiate a stable heterointerface from a precursor-to-active-phase transformation using operando spectroscopy and post-reaction mass balance.

Mesoscale architecture determines whether programmed interfaces remain functionally accessible. Dense laminates provide barrier properties and in-plane conductivity, but tortuous cross-plane transport. Hydrogels, aerogels, fibers, and printed lattices provide transport channels and tolerance to deformation. Four-dimensional printing of MXene hydrogels links stimulus-responsive shape change with pseudocapacitive storage, while multimodal hybrid hydrogels combine superelasticity and temperature sensitivity (Li et al., 2022; Liu H. et al., 2022). Nanocellulose-assisted aerogels use biomimetic texture to support both pressure sensing and compressible electrodes, showing that a single architecture can direct electrons, ions, and stress through distinct pathways (Xu et al., 2023). Architecture is thus a chemical design variable because it determines local solvent activity, ion concentration, and reaction-diffusion fields.

Porosity must nevertheless be carefully constrained. More pore volume can expose additional area but also increases oxidant access, lowers volumetric density, and disrupts conductive contacts. Mild oxidation has been used constructively to create a holey three-dimensional MXene framework with fast pseudocapacitive response (Sikdar et al., 2024). This apparent contradiction is informative because oxidation is damaging when uncontrolled, yet partial, localized conversion can be a nanofabrication tool. The key is to arrest the reaction at a reproducible intermediate state and verify that the remaining carbide network carries electrons while pores shorten ion paths. Similar logic applies to laser writing, plasma treatment, and electrochemical activation.

## 5 Function emerges from coupled transport and reaction

### 5.1 Electrochemical storage as a coupled ion–electron–strain problem

Electrochemical storage provides a stringent test of this framework because electrons, ions, solvent, strain, and redox chemistry converge at the same interface. Terminations determine ion adsorption and desolvation; interlayer species determine gallery height; flake contacts determine resistance; porosity determines diffusion length; oxidation changes all four. Flexible MXene/graphene films established that hybrid spacing can preserve conductivity while improving rapid charge storage (Yan et al., 2017). Subsequent studies moved beyond passive spacers toward chemically targeted terminal control. Nitrogen termination provides a more concrete example of a chemistry-to-function chain. In Na-ion storage, tailoring nitrogen terminals on MXene was linked to fast-charging behavior and stable low-temperature cycling, indicating that terminal atoms can regulate ion adsorption, charge transfer, and kinetic accessibility rather than simply changing the nominal composition (Xia et al., 2022). Salt-melt nitrification similarly changed terminal chemistry and improved storage response, supporting the view that terminal atoms should be evaluated through rate capability, impedance evolution, low-temperature retention, and cycling stability rather than through composition alone (Liu et al., 2023). These findings support termination-aware electrode design; however, comparisons require equal mass loading, density, electrolyte, voltage window, and oxidation history.

A frequent limitation is to equate gravimetric improvement with a superior nanoarchitecture. Open structures may show high capacity per gram while sacrificing volumetric performance and mechanical integrity. Dense films may deliver high volumetric capacitance but become rate-limited as thickness increases. The appropriate objective function depends on the device: microsupercapacitors prioritize footprint and integration, wearable electrodes prioritize deformation tolerance, and grid-scale cells prioritize mass loading and lifetime. Laser-induced MXene-functionalized graphene microsupercapacitors illustrate how local patterning, hybrid conductivity, and on-device integration can be optimized together (Deshmukh et al., 2023). Table 1 summarizes how chemical interventions propagate through structure and transport to create such functional outcomes.

**TABLE 1:** Chemistry-to-function map for surface-controlled MXene nanoarchitectures and the measurements needed to distinguish improvement from hidden failure.

Chemical intervention	Nanoscale consequence	Target function	Primary risk and decisive check	Refs
Termination exchange by halides, N-containing species, or organic ligands	Changes work function, adsorption, solvation, and gallery chemistry	Ion storage, catalysis, selective binding	Mixed or unstable terminations; quantify composition before and after operation	Liu et al. (2023), Zhang et al. (2022), Zhou et al. (2023)
Covalent or multidentate molecular/polymer ligation	Passivates reactive sites and bridges flakes	Stable conductive films, tough interfaces	Insulating overlayer or blocked pores; measure thickness-dependent transport	Lee et al. (2021), Wan et al. (2022)
Barrier matrix or sacrificial inorganic phase	Restricts oxidant diffusion or redirects reaction	Thermal and ambient stability	Protection lost during swelling; track chemical state under realistic stress	Habib et al. (2019), Liu N. et al. (2022)
Heterointerface and band/work-function alignment	Directs charge transfer and creates boundary sites	Photocatalysis, electrocatalysis, antibacterial interfaces	Reconstruction misassigned as synergy; use operando and constituent controls	Lakhan et al. (2024), Li et al. (2021)
Intercalation and controlled spacing	Balances ion access, hydration, cohesion, and electron percolation	Fast electrochemical storage	Swelling, restacking, low volumetric density; report wet spacing and loading	Děkanovský et al. (2023), Yan et al. (2017), Zhang et al. (2022)
Hydrogel, aerogel, or biomimetic fibrous assembly	Creates coupled electron, ion, solvent, and stress pathways	Flexible electrodes and multimodal sensors	Oxidant access and contact loss; cycle chemically and mechanically	Liu H. et al. (2022), Xu et al. (2023)
Controlled partial oxidation or perforation	Generates pores or oxide/carbide junctions	High-rate storage and MXene-derived active structures	Runaway conversion; map residual carbide and product distribution	Sikdar et al. (2024), Urso et al. (2022)
Printing, patterning, and polarization engineering	Fixes flake orientation, geometry, and local fields	Wireless electronics, EMI control, integrated devices	Anisotropic cracking and environmental drift; test full patterned device	Shao et al. (2022), Shepelin et al. (2021)

### 5.2 Sensing and catalysis as state-dependent interfacial reactions

Chemical sensing exploits the same variables for a different purpose. Metallic Ti_3_C_2_T_x_ gas sensors provide a concrete sensing example: adsorption-induced resistance changes on a conductive, termination-rich surface produced a high signal-to-noise response toward volatile organic compounds and gases, demonstrating that surface chemistry and electronic percolation must be evaluated together rather than separately (Kim et al., 2018). However, high response amplitude alone does not prove selectivity. Humidity interference, baseline drift, response and recovery times, and repeated-exposure stability should be reported as the measurable outcomes that determine whether the surface-state change is analytically useful. Water competes for sites, terminations alter adsorption energies, and oxidation causes baseline drift. Hybridization can add selective receptors or catalytic phases, but every added interface also creates hysteresis and diffusion barriers. Recent electrochemical phosphate sensors demonstrate that MXene interfaces can support analytically useful recognition when surface chemistry and electrode architecture are jointly controlled (Nagaraja et al., 2025). Aptasensor platforms also benefit from conductivity and ligand attachment, yet require antifouling, reference controls, and stability in biological matrices (Parihar et al., 2022). The same state-dependence becomes even more critical in catalysis, where the active interface may evolve during turnover.

Catalysis presents a more acute materials-identity problem. A pristine MXene may act as catalyst, cocatalyst, conductive support, adsorption regulator, or sacrificial precursor. In photocatalysis, MXenes can improve charge separation and provide reduction sites, but surface oxides and termination changes develop under illumination (Sun et al., 2019). For oxygen evolution, integrated MXene/metal-oxide systems can improve conductivity and create active boundaries, although the catalytic state may be reconstructed (Lakhan et al., 2024). Robust mechanistic assignments require potential-dependent spectroscopy, isotope or kinetic probes, and microscopy capable of locating transformed regions. Therefore, mechanistic analysis should focus less on whether the starting MXene is active and more on identifying the interfacial state that carries turnover under operating conditions.

### 5.3 Mechanical, electromagnetic, and environmental functions as network-level responses

Mechanical, electromagnetic, and photothermal functions are governed primarily by network topology. Conductive flakes attenuate electromagnetic waves through reflection and absorption, while heterointerfaces and pores promote polarization and multiple scattering. Waterproof multiscale composites demonstrate how dimensional hierarchy tunes impedance, loss, and environmental tolerance (Xiang et al., 2021). Direct-ink-written MXene frames add geometric control and thermochromic response (Wu et al., 2021). These properties are commonly reported as thickness-normalized shielding or reflection loss, but fair comparison also requires density, bandwidth, environmental aging, and mechanical cycling.

Mechanical measurements offer an independent assessment of chemical quality. Direct testing of Ti_3_C_2_T_x_ monolayers has clarified elastic properties and tensile strength, yet device-scale films fail through flake sliding, interfacial debonding, cracks, and oxidation rather than intrinsic sheet fracture alone (Rong et al., 2024). Tough organohydrogels use polymer-MXene interactions and solvent management to combine stretchability with environmental resistance (Yu et al., 2022). Interfacial piezoelectric polarization locking in printable MXene-fluoropolymer composites further shows that dipole alignment and charge confinement can be engineered during processing (Shepelin et al., 2021). Mechanical and electrical reliability must therefore be co-designed through the same bond network.

Environmental and biomedical functions require particular caution because transformation products interact with complex media. MXene-based remediation can combine adsorption, membrane transport, photothermal action, and catalysis, while biomedical systems exploit conductivity, optical absorption, and surface functionalization (Tunesi et al., 2021; Zamhuri et al., 2021). Trapping and detecting nanoplastics with MXene-derived oxide microrobots demonstrates that controlled conversion can generate an active mobile architecture rather than merely deactivate the parent material (Urso et al., 2022). Application claims should nevertheless distinguish performance of the initial MXene from that of transformed products and should report dissolution, oxidation, aggregation, and recovery. Descriptive toxicity alone lies outside the present scope; the chemically relevant issue is how nanoscale state evolution changes function and exposure.

## 6 A tiered validation scheme for practical MXene studies

A chemistry-first framework should not require every study to measure every possible descriptor. Instead, validation should begin with the device-limiting decision threshold. A descriptor becomes necessary only when it can explain, predict, or falsify crossing that threshold. For example, a ten percent conductivity loss may be irrelevant for an electromagnetic shield but unacceptable for a calibrated chemiresistor; a modest change in interlayer spacing may improve capacitance while causing unacceptable dimensional drift in a membrane. The purpose of state-resolved validation is therefore not to build the largest possible state vector, but to identify the smallest set of descriptors that determines the intended function and its dominant failure mode.

### 6.1 Measurement limits and uncertainty in MXene state readout

State-resolved validation must begin by recognizing that the state cannot be read out without uncertainty. XPS is necessary but not self-sufficient. Quantification of T_x_ populations depends on fitting constraints, background choice, charge correction, sputtering or beam history, and assumptions about carbide, oxide, hydroxyl, fluorine, adsorbed water, and adventitious-carbon components (Halim et al., 2016). Raman and FTIR provide useful bonding fingerprints, but their bands can overlap across terminations, adsorbed water, oxides, and organic modifiers; Raman excitation may also introduce local heating or accelerate oxidation during measurement (Adomaviciute-Grabusove et al., 2024; Parker et al., 2024). XRD gives an averaged gallery spacing rather than a complete description of swelling, staging, stacking disorder, or local interlayer chemistry, and the measured spacing can depend on humidity, cation identity, drying history, and measurement environment (Alhabeb et al., 2017; Lin et al., 2016). Microscopy resolves morphology and flake dimensions but is often statistically limited and may miss chemically distinct regions. Therefore, no single descriptor should be treated as a definitive state readout.

The practical response is not to abandon state descriptors, but to report them with method-specific limits. A defensible state assignment should combine at least one chemical probe, one structural or morphological probe, and one function-relevant measurement whenever possible (Alhabeb et al., 2017; Halim et al., 2016; Lin et al., 2016). For example, a termination claim should specify the XPS fitting model and should be checked against complementary vibrational or chemical evidence when available (Halim et al., 2016; Parker et al., 2024). A spacing claim should state whether the sample was dry, humid, solvated, or electrochemically wetted (Alhabeb et al., 2017; Lin et al., 2016). A degradation claim should distinguish oxide formation, flake fragmentation, conductivity drift, and loss of accessible surface area (Adomaviciute-Grabusove et al., 2024). In this framework, measurement uncertainty is part of the state vector rather than a nuisance hidden behind a single reported value.

### 6.2 Tier 0: synthesis and history record

Tier 0 is a synthesis-and-history record that should accompany all MXene studies, even when MXene is only one component in a composite. This record should include the precursor composition, etchant system, etching conditions, washing endpoint, delamination method, storage atmosphere, storage medium, age after delamination, batch size, and basic information on flake size and interlayer spacing (Alhabeb et al., 2017). These descriptors do not prove a mechanism, but they define whether two materials are comparable. Processing guidelines already show that etching, washing, sonication, centrifugation, storage, and flake-size control can alter yield, dispersion, oxidation, and device response (Alhabeb et al., 2017). Reporting this tier turns synthesis from a recipe into a reproducible state-preparation history.

### 6.3 Tier 1: minimal state descriptors

Tier 1 contains the minimal descriptors needed to judge whether the reported MXene state is chemically and structurally plausible for the claimed function. For most studies, this tier should include a termination or oxidation proxy, a flake-size or edge-density descriptor, an interlayer-spacing descriptor measured under relevant dry or wet conditions, and an electrical descriptor such as conductivity, sheet resistance, or contact resistance (Hart et al., 2019; Zhang et al., 2022). Morphology should also be documented sufficiently to identify restacking, pore closure, fragmentation, or phase separation. These descriptors are intentionally limited. They are not meant to capture the full complexity of the surface, but to prevent nominally identical Ti_3_C_2_T_x_ materials from being compared as though their terminations, oxidation histories, dimensions, and galleries were equivalent.

### 6.4 Tier 2: application-dependent add-ons

Tier 2 adds descriptors only when they are required by the application class. For electrochemical storage, the essential add-ons include mass loading, electrode density or thickness, wet interlayer spacing where possible, impedance or rate information, capacitance or capacity retention, and post-cycling oxidation or morphology (Perju et al., 2025; Zhang et al., 2022). For sensors, the relevant add-ons include baseline drift, humidity response, response and recovery times, selectivity controls, and signal retention after storage or repeated exposure. For catalysis, the add-ons should include post-reaction phase identification, metal dissolution or leaching where relevant, surface reconstruction, and evidence that the measured activity is not simply produced by transformed oxides or detached species (Lakhan et al., 2024). For films, coatings, and electronics, the add-ons should include sheet resistance, contact integrity, bending or humidity aging, and patterned-device performance. In this tiered scheme, the application defines which descriptors become necessary.

### 6.5 Chemistry-to-function claim rule

To avoid generic coupling claims, a chemistry-to-function argument should specify three elements: the controlled variable, the measured state change, and the measured functional outcome. For example, changing the etchant is meaningful only when it is linked to measured spacing, morphology, resistance, or capacitance; termination exchange is meaningful only when surface chemistry is connected to rate capability, work function, adsorption, or stability; and oxidation control is meaningful only when oxide formation, conductivity drift, or structural fragmentation is tracked under defined exposure conditions.

### 6.6 Tier 3: mechanism-testing descriptors

Tier 3 is required only when a study claims a causal mechanism rather than reporting a practical performance improvement. If termination exchange is claimed to improve storage, samples should preferably originate from one parent batch and differ primarily in the exchange treatment (Hart et al., 2019; Zhang et al., 2022). Electrolyte, loading, thickness, and oxidation history should be held constant. If an interface is claimed to improve catalysis, the hybrid should be compared with both constituents, a physical mixture, and, where possible, an interface-disrupted control (Lakhan et al., 2024). If a barrier is claimed to suppress oxidation, oxygen and water permeability should be separated from intrinsic reaction rate. Operando characterization, mass balance, factorial designs, and device-scale transport models are therefore not universal requirements; they are mechanism-testing tools that become necessary when the claim depends on separating coupled variables (Lakhan et al., 2024; Perju et al., 2025).

### 6.7 Stopping rules, benchmark materials, and round-robin validation

A practical validation scheme also needs stopping rules. Once the descriptor that controls the device-limiting threshold has been identified, additional characterization should be justified by its ability to change the interpretation. For a conductor, a decisive check may be whether sheet resistance remains within the allowable range after humidity and bending. For a supercapacitor, it may be whether capacitance retention can be linked to wet spacing, impedance, and oxidation after cycling (Perju et al., 2025; Zhang et al., 2022). For a chemiresistor, it may be whether baseline drift and humidity interference exceed the calibration window. For a catalyst, it may be whether the active phase after operation is still the starting MXene, a reconstructed interface, or an oxide-derived product (Lakhan et al., 2024). This approach preserves the rigor of state-resolved validation while making it usable: every study reports a common minimal history, application studies add only the descriptors needed for their target function, and mechanistic studies add controls only when they are required to falsify the proposed chemistry-to-function chain. Shared spectral libraries, raw spectra, and consistent fitting protocols can further reduce assignment drift across laboratories (Parker et al., 2024).

Because no certified reference MXenes are yet available, field-level validation should also move toward shared benchmark dispersions and films. A useful round-robin exercise would distribute the same MXene batch to multiple laboratories and compare XPS fitting outputs, Raman and FTIR assignments, XRD spacing under defined humidity or electrolyte conditions, flake-size statistics, conductivity, and storage-dependent drift. The goal would not be to force one universal fitting model or one universal synthesis route. Rather, it would establish realistic uncertainty ranges for nominally identical samples and reveal whether discrepancies arise from synthesis, storage, characterization, data fitting, or device testing. Shared spectral libraries, raw spectra, fitting constraints, and benchmark materials would make state-resolved descriptors more comparable across laboratories (Parker et al., 2024).

## 7 Design rules, controversies, and research priorities

### 7.1 State-first design

The first design rule is to define the target interfacial state before selecting a synthesis route (Hart et al., 2019; Zhang et al., 2022). If an application requires strong covalent coupling, a surface rich in–OH or–O groups may be more useful than a nominally high-conductivity, –F-rich surface (Lee et al., 2021). If fast ion transport is dominant, gallery solvation and reversible ion coordination may matter more than dry interlayer spacing (Zhang et al., 2022). If ambient electronics are targeted, edge protection and barrier architecture may outweigh maximum initial conductivity (Habib et al., 2019; Iqbal et al., 2021; Natu et al., 2019). Reversing the conventional workflow in this way, from desired reaction and transport to synthesis, prevents the common practice of applying one Ti_3_C_2_T_x_ stock dispersion to unrelated devices (Hart et al., 2019; Iqbal et al., 2021).

Operationally, this state-first design can be translated into several practical routes. A surface enriched with–O and–OH groups can be approached through controlled post-etch washing, mild oxidative conditioning, or covalent ligand coupling, provided that conductivity and oxide growth are monitored. A surface enriched with halogen or nitrogen terminals can be pursued through Lewis-basic halide treatment or salt-melt nitrification when changes in spacing, termination chemistry, and impedance are tracked together. If ambient stability is the dominant target, polymeric ligation, block-copolymer or elastomeric passivation, and inorganic barrier phases can be introduced immediately after delamination under low-oxygen or low-temperature conditions. In each case, the synthetic route should be paired with an endpoint descriptor, such as XPS-derived termination/oxidation state, wet interlayer spacing, flake-size distribution, sheet resistance, or storage-dependent drift.

### 7.2 Designed heterogeneity and edge-selective chemistry

The second rule is to design gradients rather than demand uniformity everywhere. Basal planes can provide conduction, edges can host selective chemistry, outer layers can provide protection, and interior galleries can regulate transport. The field often treats chemical heterogeneity as an impurity, yet controlled heterogeneity enables division of labor. High-entropy carbide MXenes are relevant to designed heterogeneity not simply because they introduce bulk compositional complexity, but because metal-sublattice disorder is expected to create distributions of local M–T_x_ bond strengths, termination affinities, water/oxygen adsorption energies, and edge oxidation barriers (Nemani et al., 2021). In this view, the metal sublattice becomes an upstream chemical handle for programming surface and edge reactivity. However, high-entropy composition alone should not be treated as proof of functional heterogeneity; it should be linked to spatially resolved termination, oxidation, or adsorption measurements. The central challenge is to distinguish designed gradients from uncontrolled batch variation. Spatially resolved spectroscopy and microscopy, combined with statistically meaningful flake populations, are therefore needed.

A practical route to designed heterogeneity is to build selectivity through the processing sequence rather than by demanding atomically perfect patterning. Basal-plane protection can be attempted by adsorbing or grafting polymeric ligands before drying (Lee et al., 2021), whereas edge-enriched chemistry can be tested by combining flake-size fractionation with selective surface modification or labeling at more reactive edge/defect sites (Maleski et al., 2018; Wang et al., 2016). Outer-layer protection can be introduced by barrier polymers or inorganic shells, while interior-gallery transport can be tuned through intercalants, molten-salt/halide treatments, or zwitterionic molecules (Děkanovský et al., 2023; Hart et al., 2019; Kamysbayev et al., 2020). Controlled topochemical transformations, including mild oxidation or laser-induced local patterning, provide another route when the goal is to generate holey sheets, locally converted regions, or oxide/carbide junctions rather than preserve the pristine carbide everywhere (Deshmukh et al., 2023; Sikdar et al., 2024).

Edge-selective chemistry is particularly important because basal-plane averages can hide the sites that control degradation, recognition, and catalytic response (Iqbal et al., 2021). Edges contain under-coordinated atoms, etching damage, and termination motifs that differ from extended terraces (Natu et al., 2019). They can accelerate oxidation while potentially increasing the density of chemically reactive sites, creating a stability-selectivity trade-off (Natu et al., 2019; Xia et al., 2019). Future experiments should compare flakes with controlled lateral size at similar thickness and basal chemistry, then use selective passivation or labeling to test whether the response scales with edge density (Maleski et al., 2018). This distinction is essential for translating small-flake laboratory dispersions into large-flake films and patterned devices.

### 7.3 Operando validation and unresolved controversies

The third rule is to characterize state during operation. *Ex situ* measurements can mistake a precursor for the active material or miss transient swelling, redox, and interfacial reconstruction (Lin et al., 2016). Operando or *in situ* diffraction can track gallery changes (Lin et al., 2016), Raman and infrared spectroscopy can follow bonding and degradation pathways (Adomaviciute-Grabusove et al., 2024), X-ray photoelectron methods can resolve metal and termination states (Halim et al., 2016), and coupled electrochemical measurements can connect resistance, thickness, and mass changes to charge storage (Perju et al., 2025). No single technique can provide a complete state vector. Multimodal measurements should therefore be synchronized with conductivity, catalytic rate, sensor baseline, or mechanical response so that a chemical transition can be connected to a functional transition rather than merely correlated after failure (Perju et al., 2025).

Three controversies require direct experimental tests. First, the conductivity-termination relationship is often generalized from calculations or dissimilar samples; controlled termination exchange on the same parent batch is needed (Hart et al., 2019). Second, apparent anti-oxidation effects may arise from reduced oxygen diffusion rather than intrinsically stable surfaces; permeability and reaction kinetics should be separated (Liu N. et al., 2022). Third, hybrid synergy is frequently claimed without additive controls at equal loading and morphology. A convincing study should compare the hybrid with both constituents, a physical mixture, and an interface-disrupted control, especially because MXene-based heterointerfaces may reconstruct under electrocatalytic conditions (Lakhan et al., 2024). Such experiments would be more informative than another record value obtained under unique conditions.

### 7.4 Scalability, data standards, and reporting requirements

Scalability introduces additional chemical constraints because scale-up changes the reaction environment rather than merely increasing reagent volume (Lakhe et al., 2019; Lim et al., 2022). Fluoride-lean and molten-salt routes broaden composition and may reduce some hazards, but they introduce questions related to salt purification, corrosion, energy use, reagent recovery, and termination control (Arole et al., 2021; Li et al., 2020). During scale-up, concentration and temperature gradients, gas exchange, washing efficiency, and shear history may change relative to small-batch synthesis. These factors can alter etching completion, defect density, intercalation, flake size, and oxidation before the product reaches storage (Alhabeb et al., 2017). A credible scale-up study should therefore sample multiple reactor positions and batches, report both yield and chemical-state distributions, and test whether downstream films or electrodes retain their target performance (Alhabeb et al., 2017). Continuous or semicontinuous processing may improve control, but only when residence time and reagent recovery are linked to termination and defect outcomes.

Improved data practices are equally important for turning programmable interfaces into predictive nanoscience. A useful MXene record should pair synthesis metadata with machine-readable termination fractions, oxidation indicators, flake-size distributions, interlayer spacing under relevant humidity or electrolyte, storage history, and performance conditions. The benchmark-material and round-robin strategy proposed in Section 6 would further support scalable data standards by defining realistic uncertainty ranges for MXene state descriptors (Parker et al., 2024).

A minimum reporting standard need not be burdensome. Authors could provide one synthesis-and-history table, one state characterization panel, and one application-specific stress test. The table would record precursor, etchant, washing, delamination, storage, age, and batch size (Alhabeb et al., 2017). The characterization panel would quantify terminations, oxidation, dimensions, and spacing, while the stress test would connect chemistry to the device-limiting metric. Raw spectra and fitting constraints should be deposited when possible (Parker et al., 2024). This reporting package would not eliminate methodological diversity, nor should it. Instead, it would make diversity interpretable, enabling meta-analysis across laboratories and preventing nominally identical MXenes from being compared as though their surfaces, galleries, and degradation histories were the same.

## 8 Conclusion

MXenes are best viewed as reactive nanoarchitectures whose identity evolves from etching through operation. Surface terminations, defects, intercalants, water, oxidation products, and binding partners form a coupled chemical state that controls electronic structure, colloidal behavior, assembly, transport, mechanics, and reaction pathways. This perspective unifies observations that otherwise appear application-specific. A stabilizing ligand also changes ion solvation, a polymer bridge affects tunneling and diffusion, and an accessible pore can admit oxidants. Likewise, a catalytic heterointerface may reconstruct into a different active phase.

The practical consequence is a transition from performance-first screening to state-first design. Researchers should define the required surface reaction, prepare and report the corresponding chemical state, assemble interfaces with a specified transport role, and verify state evolution under realistic stress. Table 1 translates this sequence into testable choices and failure checks. Recent advances in termination exchange, covalent ligation, controlled oxidation, printed structures, hydrogels, and operando pathway monitoring show that the required tools already exist (Adomaviciute-Grabusove et al., 2024; Lee et al., 2021; Li et al., 2022; Zhou et al., 2023). The next priority is to integrate them through common descriptors and controls.

For nanoscale materials chemistry, MXenes offer a chemically rich testbed in which synthesis, assembly, characterization, and function are inseparable. Progress will depend on reproducible state preparation, fluorine-lean scalable chemistry, spatial and operando characterization, and comparisons that expose rather than hide trade-offs. Meeting these requirements will allow MXenes to move from versatile but variable flakes toward programmable platforms whose stability and function can be predicted from interfacial chemistry.

A practical near-term strategy is to organize studies around one falsifiable chemistry-to-function chain: identify the intended surface state, verify that state after processing, quantify the transport pathway it is expected to alter, and test the dominant failure mechanism under operation. Reporting boundary conditions and negative outcomes will be as valuable as reporting record values because they define where a termination, ligand, or architecture ceases to work.
